# Exploring Within-Gender Differences in Friendships Using an Online Social Network

**DOI:** 10.1007/s10508-024-02906-5

**Published:** 2024-06-11

**Authors:** Pietro Pollo, Tania A. Reynolds, Khandis R. Blake, Michael M. Kasumovic

**Affiliations:** 1 Evolution and Ecology Research Centre, School of Biological, Earth and Environmental Sciences, University of New South Wales, 5 Floor, Building E26, Kensington, NSW 2052 Australia; 2 Department of Psychology, University of New Mexico, Albuquerque, NM USA; 3 Melbourne School of Psychological Sciences, The University of Melbourne, Melbourne, VIC Australia

**Keywords:** Body image, Evolutionary psychology, Female competition, Friendship preference, Intra-gender competition, Mating rivalry

## Abstract

People tend to befriend others similar to themselves, generating a pattern called homophily. However, existing studies on friendship patterns often rely on surveys that assess the perspective of relatively few participants on their friendships but do not measure actualized friendship patterns. Here, we used data from a large Slovakian online social network to assess the role of gender, age, and body mass index (BMI) in same-gender online connections among more than 400,000 users. We found that age and BMI homophily occurred in both men’s and women’s same-gender connections, but somewhat more strongly among men’s. Yet, as women diverged in BMI, their connections were less likely to be reciprocated. We discuss how the evolutionary legacy of men’s coalitional competition (e.g., warfare) and women’s mating competition or recruitment of allocare providers might contribute to these patterns in modern same-gender relationships. For example, men’s engagement in physical activities may lead to similar formidability levels among their same-gender peers. Altogether, our findings highlight the importance of trait similarity to same-gender friendship patterns.

**Supplementary Information:**

The online version contains supplementary material available at 10.1007/s10508-024-02906-5.

## Introduction

Friendships are social bonds that can strongly influence fitness across several species (Brent et al., 2014; Silk et al., 2003). For humans, friendships represent social contracts that are expected to provide mutual benefits (Vigil, 2007), such as companionship, emotional support, help in time of need, self-disclosure, and even alliances during conflict (Argyle & Henderson, 1984; DeScioli & Kurzban, 2009; Hall, 2011, 2012). Ultimately, friendships contribute to human happiness as they can fulfil several psychological needs (Demir & Özdemir, 2010). Yet, friends demand limited resources like time, cognitive capacity, and preferential treatment (Barakzai & Shaw, 2018; Dunbar, 2018; Powell et al., 2012), thereby constraining the size of friendship networks (Hill & Dunbar, 2003). Given these upper limits on the quantity of friends in whom individuals can invest, humans should carefully select their friends in a process attuned to maximize their own fitness and goals (Krems & Conroy-Beam, 2020).

Many studies delve into individuals’ friendship expectations by asking participants about their preferred characteristics in friends (e.g., Ayers et al., 2023; Hall, 2011, 2012; La Gaipa, 1987). These studies indicate that traits reflecting the capacity to support others are generally favored in potential friends (Hall, 2012). For example, people prefer to bond with individuals who are kind, trustworthy, and loyal, probably because these qualities represent willingness to reciprocate (Ayers et al., 2023; DeScioli & Kurzban, 2009; Vigil, 2007). However, individuals also vary in their friendship preferences and the standards to which they hold their friends (Krems & Conroy-Beam, 2020).

If friendship expectations vary among individuals, factors that determine actual friendship formation and maintenance are likely to be even more complex. This is because individuals can only befriend others when they have opportunities to do so, such as sharing a common language (Curry & Dunbar, 2013) or a social space (Back et al., 2008). In other words, the likelihood of two individuals becoming friends may depend on certain expectations, but also on the situation and characteristics they share. Indeed, people tend to bond with similar individuals, a pattern called homophily (McPherson et al., 2001).

Homophily has been observed for demographic characteristics, such as race (Moody, 2001), socioeconomic status (Chabot, 2024), and religion (Smith et al., 2014). Even though homophily can arise from external factors like physical proximity, it can also originate from personal preferences. For example, individuals show preferences for those who wear similar clothing to themselves (Back et al., 2011). Consequently, individuals often exhibit similar behaviors, attitudes, values, and personalities to their friends (e.g., DeScioli & Kurzban, 2009; Harris & Vazire, 2016; Lönnqvist & Itkonen, 2016; Mehlman, 1962; Urberg et al., 1998). Moreover, same-gender friendships are more frequent and stronger than opposite-gender ones despite an equal ratio of men and women in the population (e.g., Bhattacharya et al., 2016; Laniado et al., 2016; Palchykov et al., 2012). Gender homophily could result from long-standing cultural forces, such as division of labor based on gender (e.g., Marlowe, 2007; but see Anderson et al., 2023; Lacy & Ocobock, 2024; Ocobock & Lacy, 2024). Ultimately, preferences for similar others are likely to occur because trait similarity may ease communication, promote cooperation (Melamed et al., 2020), and even reflect underlying genetic similarity (Fowler et al., 2011). Hence, emotional closeness increases as friends share more characteristics (Curry & Dunbar, 2013; but see Block & Grund, 2014), suggesting that trait similarity is a critical factor in the initiation and maintenance of friendships.

Evidence suggests an overall tendency toward homophily yet these inclinations might differ among cis heterosexual men and women (hereby simply men and women). This is because men and women are under distinct social pressures (Kimmel & Aronson, 2013) and have been so across human evolutionary history (Buss, 2019), which we discuss below.

### Factors That Can Affect Friendships Among Women

Women’s appearance influence their success in mating competition, as men exhibit stronger preferences than do women for mates who are young (Maestripieri et al., 2014; Mathes et al., 1985; Matts et al., 2007; McLellan & McKelvie, 1993) and physically attractive (Bar-Tal & Saxe, 1976; Buss, 1989; Townsend & Wasserman, 1998; Walter et al., 2020). Compared to their heavier counterparts, thinner women are perceived as more physically attractive in many cultures (Kurzban & Weeden, 2005; Perilloux et al., 2013; Weeden & Sabini, 2007) and are more likely to attract mates with resources (Averett et al., 2008; Oreffice & Quintana-Domeque, 2010).

Women are not passive in the mating market, but actively compete with other women for mates (Fisher, 2013), which affects their responses to their same-gender peers. Women compare their bodies to one another (Jones & Buckingham, 2005) and experience body dissatisfaction when exposed to thinner women (Krones et al., 2005). Compared with men, women feel more distressed by same-gender rivals who have more attractive bodies (Buss et al., 2000), such as those with narrow waists relative to their hips (Fink et al., 2014). Although women express preferences for friends who surpass them in many attributes, physical attractiveness is one exception, such that women generally prefer friends of similar attractiveness (Vigil, 2007). When female friends diverge in physical attractiveness, the less attractive friend detects greater mating rivalry within the friendship (Bleske-Rechek & Lighthall, 2010; Bleske-Rechek et al., 2014), highlighting the value assigned to relative appearance differences. Not only do women feel threatened by physically attractive same-gender peers, they also report a stronger likelihood of deceiving and disparaging them more relative to less physically attractive women (Fink et al., 2014; Reynolds et al., 2018).

Because women’s age and body size correlate with their attractiveness (Kościński, 2013; Maestripieri et al., 2014; Mathes et al., 1985; Matts et al., 2007; McLellan & McKelvie, 1993; Perilloux et al., 2013; Swami & Tovée, 2005; Tovée & Cornelissen, 2001; Tovée et al., 1998; Weeden & Sabini, 2005, 2007), it is possible that larger divergences in these traits among female peers may generate greater tension and reduce the likelihood of reciprocated friendships. Indeed, there is some evidence that girls are more homophilous in body size than are boys (Badaly, 2013), but data are scarce for adults.

Although same-gender peers can affect women’s prospects for attracting mates, they might have also offered benefits in the form of allocare, support, aid, and information across our evolutionary history (Hess & Hagen, 2002; Hrdy, 2009; Reynolds, 2022). For example, across non-industrialized groups, women receive childcare assistance mostly from genetic kin, especially from their mothers (i.e., children’s maternal grandmothers; Crittenden & Marlowe, 2008; Ivey, 2000; Page et al., 2019; Wood & Eagly, 2002). Younger girls also provide allocare, perhaps as a form of practice for their later reproductive efforts (Lancaster, 1971). If these patterns in non-industrialized societies mirror those of ancestral women, then female ancestors may have benefitted from maintaining cross-generational relationships with other women, and perhaps especially with family members. Indeed, compared to men, modern women maintain greater contact with cross-generational family members (David-Barrett et al., 2016; Sneed et al., 2006). These pressures to recruit reliable and trustworthy alloparents may have reduced age-based homophily in women’s same-gender relationships.

### Factors That Can Affect Friendships Among Men

Across human history, men were more often engaged in physically demanding and risky coalitional activities, including large-game hunting and warfare than were women (Geary, 2021; Marlowe, 2007; Wrangham, 1999). Groups composed of men with traits advantageous for these pursuits should have been more likely to secure necessary calories and emerge victorious on the battlefield. These pressures have likely contributed to the physical and mental traits modern men possess today (Hill et al., 2013; Kordsmeyer et al., 2018; Puts, 2023). Because these competitions were group-based, men could enhance their own fitness by cooperating with other men who possessed attributes that enhanced the coalition’s success. Such historical selective pressures might at least partially explain modern men’s preferences for same-gender friends’ courage and group loyalty (Krems & Neuberg, 2022; Winegard et al., 2016). Coalitionary members’ physical attributes, such as physical strength, may have also affected the likelihood of group success (Apicella, 2014). For instance, compared to women, men show stronger preferences for same-gender friends’ physical strength (Eisenbruch et al., 2016), athleticism (Hall, 2011; Reynolds & Palmer-Hague, 2022; Vigil, 2007), and ability to “back them up” in physical conflicts (Williams et al., 2022). Even in modern societies, boys and men are much more likely than girls and women to participate in all sports, but especially in hunting and combat sports (Deaner & Smith, 2013). Indeed, adolescent boys show stronger similarity to their peers on physical activity than do girls (Badaly, 2013). Altogether, these patterns might contribute to body size homophily in men’s same-gender friendships.

Besides valuing physical strength, groups of men could have also capitalized on the divergent skillsets of their component members to enhance the likelihood of success. For example, men’s hunting prowess tends to peak around age 40, which is much later than men’s peak physical strength (Gurven et al., 2006). A group aiming to capitalize on men’s age-related patterns of acquired skill and physical strength would comprise both younger and older men, decreasing age homophily in men’s same-gender friendships.

### Present Study

Many studies examining friendship preferences and patterns are conducted using surveys (e.g., DeScioli & Kurzban, 2009; Krems & Conroy-Beam, 2020; Migliaccio, 2009; Reynolds & Palmer-Hague, 2022; Vigil, 2007; Williams et al., 2022). Although this type of approach yields high-quality data, sample sizes are often small and constrained to specific demographic groups (e.g., white college students from the United States), which can misrepresent average behavioral patterns across all humans. In addition, surveys usually explore participants’ ideal or existing friendships exclusively from the participants’ perspective, thereby neglecting friendship properties that stem from the other individual (e.g., whether the friendship is reciprocated). To circumvent these issues, we use data from a Slovakian online social network, called Pokec, to explore gender differences in homophily patterns across a large population of individuals. Pokec users were able to befriend one another and could share personal information, including gender, age, and body measurements. Following another user did not require being followed back, allowing us to examine whether trait similarity influenced reciprocity rates.

Here, we explored gender differences in online friendship homophily regarding age and body mass index (BMI) as these traits can influence various aspects of people’s lives in modern (e.g., mate attraction: Wang et al. 2015; social dominance: Swami et al., 2013) and ancestral contexts (see subsections above). Even though reasonable hypotheses could be generated about both men’s and women’s inclination toward BMI-based homophily in their same-gender relationships, we leave the examination of gender differences in the magnitude of these tendencies as exploratory. Because opposite-gender friendships can involve romantic and sexual intentions (Bleske-Rechek et al., 2016; Lewis et al., 2012; Szymkow & Frankowska, 2022), we investigated only same-gender relationships (although some might be of romantic nature) in our study. We also note that gender and sex are distinct concepts, with the former related to individuals’ performance and identity regarding societal norms (Morgenroth & Ryan, 2018), while the latter is a biological designation based on gametes, genitals, chromosomes, or hormones (McLaughlin et al., 2023). We use the term gender throughout our manuscript because Pokec users were specifically asked about their gender identity, so we have no way to ascertain whether these classifications reflect biological sex. Although gender is not binary (Lindqvist et al., 2021), we classified users as either “women” or “men” because these were the options available on the Pokec platform.

## Method

### Subjects

We used data from Pokec, a popular Slovakian online social network, extracted by Takac and Zabovsky (2012) (publicly available at Stanford University repository). This dataset contains information for more than 1.6 million users, including their online ties with each other. Gender, age, and body measurements were self-reported. To reduce potentially erroneous data, we treated data points as missing if they were unlikely to be true using arbitrary but intuitive thresholds (i.e., age lower than 12 or greater than 59, height lower than 140 cm or greater than 200 cm, body mass lower than 10 kg or greater than 200 kg, BMI lower than 15 or greater than 35). In combination with pre-existing missing data, this process excluded 0.1% of users (nodes) for unknown gender, 32.9% for unknown age, and 65% for unknown BMI. Altogether, we had information for all of these three attributes for a little more than a quarter of users in the network (25.6%, approximately 419,000 users).

Given that age was substantially skewed (Figure S1), we analyzed data for young adults only (i.e., users from 15 to 32 years old). This age interval represented approximately one standard deviation from the median (*x̅* = 24.7 years old; *M* = 23; *SD* = 8.6) and included 76.4% of users with known age (Fig. S1). Analyzing only young adults allowed us to explore a more homogeneous sample of the network. Moreover, because individuals in this age interval are often involved in mating competition and reproduction, then stronger inferences could be made regarding the potential pressures on same-gender relationships related to these motives.

### Measures and Statistical Analyses

We used several approaches to measure homophily in the Pokec network. First, we used Newman’s assortativity coefficient (Newman, 2002, 2003) to compare whether segregation in age and BMI for each gender was greater than expected by chance. This metric is equivalent to a Pearson’s correlation between users and their followers for a given attribute. It ranges from − 1 to 1, indicating whether ties occur between nodes with the opposite or the same value of that attribute, respectively. An assortativity coefficient of zero value represents no segregation (i.e., ties between similar and dissimilar nodes occur with the same frequency). We obtained assortativity coefficients for different subsets of the network to test gender differences and to ensure the robustness of our inferences. That is, we divided the network by gender and calculated age and BMI assortativity in each to explore whether homophily for these traits differed among women’s versus men’s same-gender ties. Additionally, we used age and BMI intervals to control for possible correlations between these traits. For instance, if friendships are dependent on similarity only in age or only in BMI (but not both), we could still find homophily for the “irrelevant” trait if age and BMI are correlated. To circumvent this issue, we calculated assortativity coefficients in subsets of the network containing only users of the same interval, controlling for the other trait.

Homophily can arise from opportunity, so it is crucial to calculate the homophily that can occur in the network simply by chance. To do this, following Laniado et al. (2016), we shuffled nodes’ attributes (i.e., gender, age, and BMI) among users that had the same in- and out-degree (i.e., number of followers and number of followees, respectively), producing shuffled networks with the original structure. We simulated this process 100 times and compared the mean assortativity coefficient of these shuffled networks with the assortativity coefficient obtained from the network in its original state, deeming them as different from one another if the observed value was not contained in the 95% confidence interval of the former.

One issue with assortativity coefficients is that they cannot be statistically compared to one another as they are single values (i.e., they are metrics without an error estimate). To circumvent this issue and complement our results, we fitted two generalized linear mixed models (GLMMs) to Pokec’s data. These models followed the same rationale as assortativity coefficients: greater segregation for a given attribute in a network should lead to a stronger association between users and the ones they follow regarding that attribute. Therefore, these models essentially correspond to assortativity coefficients, but allowed us to statistically compare the degree of similarity in friendships among women with the degree of similarity in friendships among men. We only used reciprocal ties (i.e., connections between users that followed one another online) in these models as these ties should show greater similarity, and also because we assessed effects on reciprocity in a separate analysis (see below).

We ran one GLMM for age and the next for BMI (Models 1 and 2 respectively). Both GLMMs were conducted on the data in long format, such that each row represented a follower-followee dyad, and each user could be represented multiple times (depending on how many friendships they had). In the age GLMM, the followee’s age served as the outcome variable, whereas the follower’s age and the gender of the followee/follower dyad (i.e., either men or women) were the predictor variables. We also added the difference in BMI between the followee and follower (i.e., followee’s BMI subtracted from the follower’s BMI), all possible interactions as covariates, and the identity of followers as random intercepts in the model. For the BMI GLMM, we ran the same model but substituted age with BMI (and vice versa).

The analyses above explored whether segregation in age and BMI occur in same-gender online ties. However, they did not explicitly evaluate whether similarity in these traits was associated with tie reciprocity, which we interpret as a measure of willingness to initiate or maintain a connection. To investigate tie reciprocity, we fitted a third GLMM to the dataset, but this time with a binomial error structure and a logit link function (Model 3). In this model, tie reciprocity (non-reciprocal as 0, reciprocal as 1) was the outcome variable, while predictor variables were the absolute differences in age and BMI between follower and followee, the gender of the dyad, and all interactions.

All statistical analyses were conducted in the software R 4.0.3 (R Core Team, 2022). Assortativity coefficients were obtained using the function *assortativity* from the package *igraph* (Csárdi & Nepusz, 2006). We fitted GLMMs using the functions *lmer* and *glmer* from the package *lme4* (Bates et al., 2015), using the optimizer *bobyqa* from the package *minqa* (Bates et al., 2014) to improve convergence. All continuous predictor variables were standardized before model fitting, as well as response variables in the first two models. All data (both original and processed) and codes have been made publicly accessible (see Declarations section below).

## Results

### Overview of the Network

**Fig. 1:**
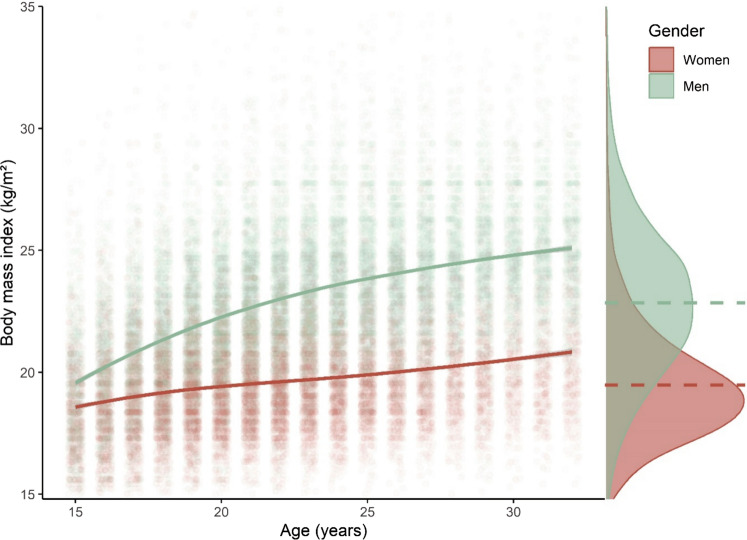
Relationship between age and body mass index (BMI) for each gender in users of the Pokec network. Curves represent trends in the data, with shaded areas representing its 95% confidence interval. Density plots represent BMI distributions, in which dashed lines represent mean values

We found a strong gender difference regarding among users’ BMI, as men’s mean BMI (*x* = 23 kg/m^2^) was much greater than women’s mean BMI (*x* = 19.6 kg/m^2^; Cohen’s *d* = 1.22; density plots on Fig. 1). BMI increased with age, but more strongly for men than for women (Spearman correlation: rho_men_ = 0.49, rho_women_ = 0.24, both *p* < 0.001; Fig. 1).

### Age Segregation

**Fig. 2:**
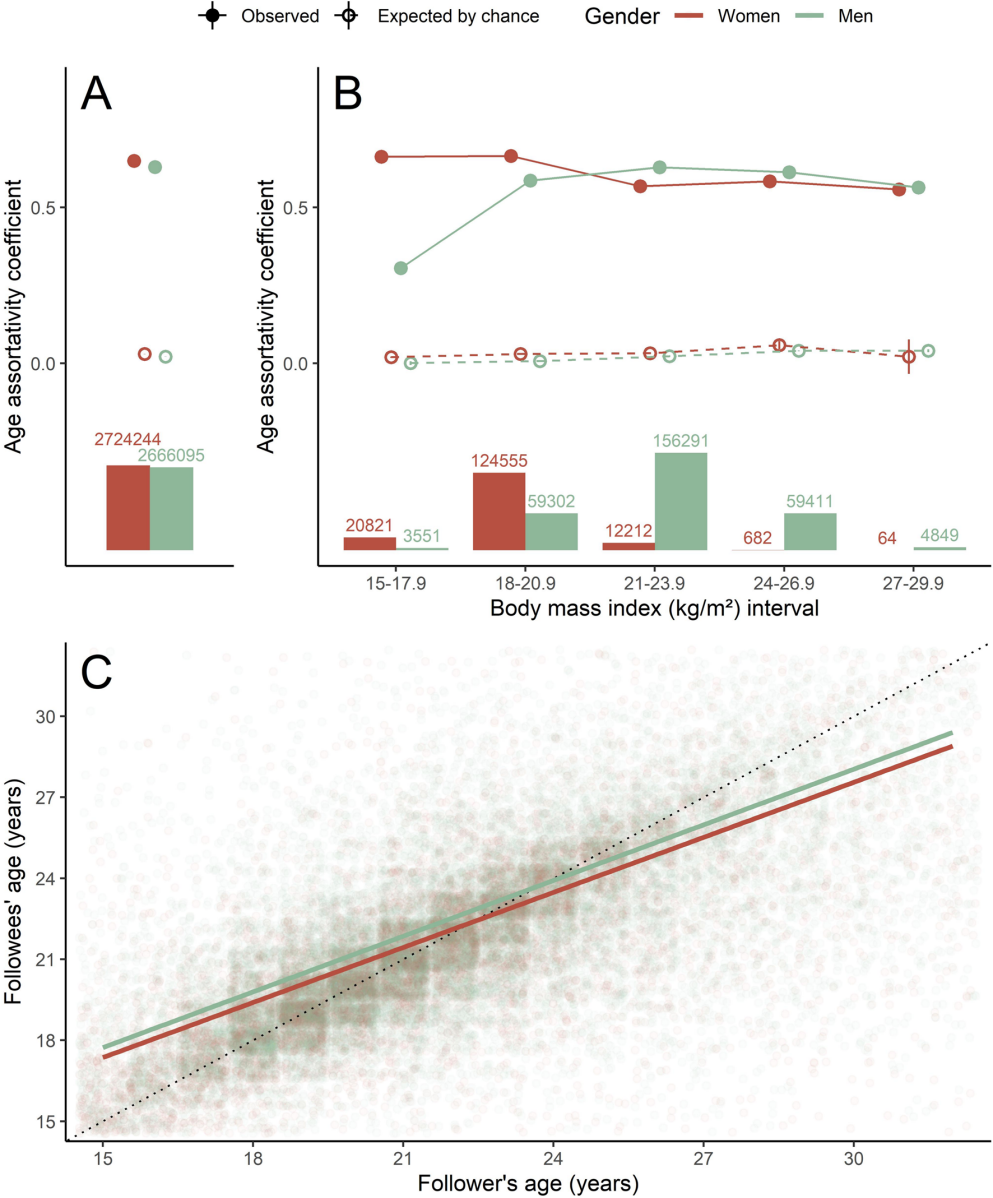
Age segregation for each gender. **A** Age assortativity coefficients observed (filled dots, continuous lines) and expected by chance (empty dots, dashed lines) in the network (**A**) and in subsets of the network using certain BMI intervals. Bars represent the number of observed connections in the network (**A**) and in each of its subsets (**B**). Whiskers represent the 95% coefficient interval for mean assortativity coefficients (only for the ones expected by chance). **C** Results of a GLMM that assess the average age relationship between users in the network. The dotted line indicates a perfect relationship between the axes, present for comparison purposes. Numbers above the bar graphs are sample sizes

The age assortativity coefficient for women and men were similar (*r*_women_ = 0.65, *r*_men_ = 0.63), and both were greater than expected by chance for women (*r*_women_ = 0.03) and men (*r*_men_ = 0.02; Fig. 2A). When considering only connections among users with similar BMI, assortativity coefficients were all greater than expected by chance in each interval. However, differences between men and women regarding these coefficients were inconsistent (Fig. 2B), regardless of how we subsetted BMI intervals (Fig. S2). We also could not obtain assortativity coefficients when BMI exceeded 29 as there were very few individuals with these BMI values in the network. According to our GLMM that controlled for BMI differences between users (Model 1), the age relationship between same-gender users in reciprocal connections (i.e., following each other) was slightly stronger among men than among women (*β*_men_ = 0.65, *β*_women_ = 0.64, *p* < 0.001; Fig. 2C). That is, men tended to more frequently associate with similarly aged peers than did women by a very small degree.

### Body Mass Index Segregation

**Fig. 3:**
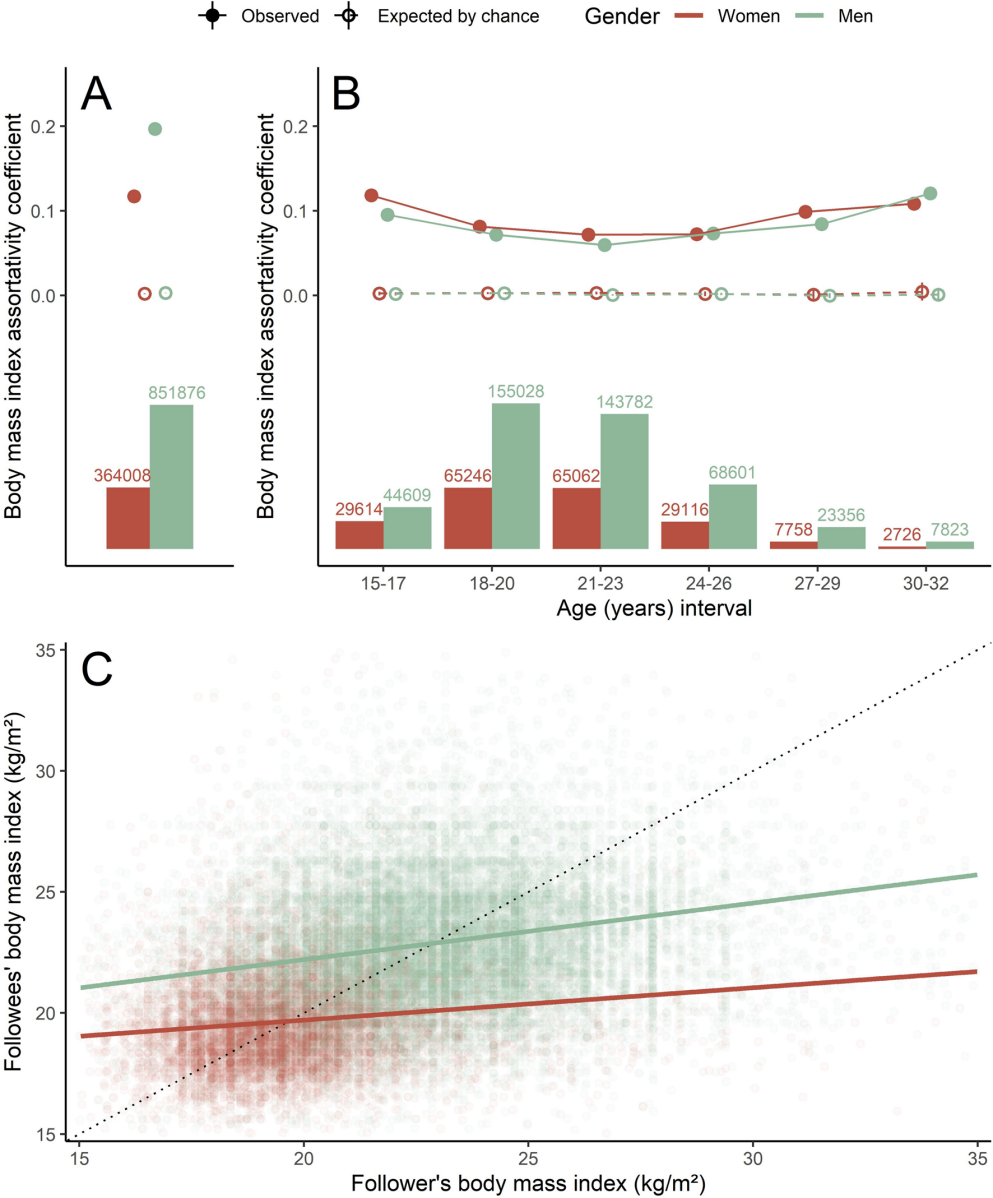
Body mass index (BMI) segregation for each gender. BMI assortativity coefficients observed and expected by chance in the network (**A**) and in subsets of the network using certain age intervals (**B**). Bars represent the number of observed connections in the network (**A**) and in each of its subsets (**B**). Whiskers represent the 95% coefficient interval for mean assortativity coefficients (only for the ones expected by chance). (**C**) Results of a GLMM that assess the average BMI relationship between users in the network. The dotted line indicates a perfect relationship between the axes, present for comparison purposes. Numbers above the bar graphs are sample sizes

The BMI assortativity coefficient for men was greater than for women (*r*_men_ = 0.20, *r*_women_ = 0.12), and both were greater than expected by chance (both *r* = 0; Fig. 3A). When considering only ties among people of similar age (i.e., in the same age interval), the BMI assortativity coefficient was always greater than expected by chance. However, differences between men and women regarding these coefficients were not consistent (Fig. 3B), regardless of how we subsetted age intervals (Figures S3). When we controlled for age differences between users (Model 2), the BMI relationship between same-gender users in reciprocal connections (i.e., following each other) was stronger among men than among women (*β*_men_ = 0.23, *β*_women_ = 0.13, *p* < 0.001; Fig. 3C). That is, men more often formed connections with similar BMI peers than did women.

### Reciprocity

**Fig. 4:**
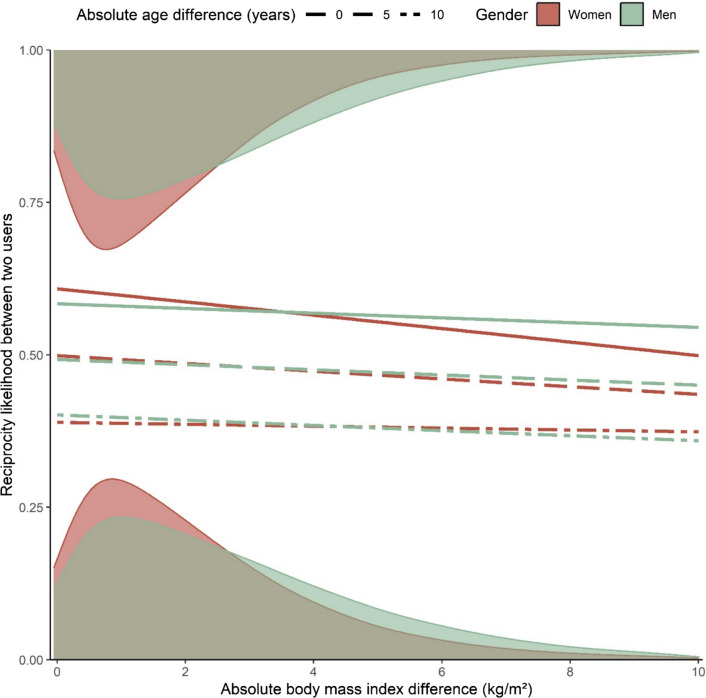
Reciprocity likelihood between two users depending on their gender and their absolute differences in body mass index (BMI) and age. Density plots represent the relative amount of reciprocal (top) and non-reciprocal (bottom) connections in the network

Reciprocity likelihood (i.e., mutual following opposed to only one user following another) decreased with absolute difference in both age and BMI for both genders (Fig. 4). The effect of absolute age difference was similar in magnitude for men and women (*β*_women_ = − 0.19, *β*_men_ = − 0.17, *p* = 0.08), suggesting men and women were similarly likely to form reciprocated connections with same-aged peers. Conversely, increasing the absolute BMI difference between two individuals of the same age from zero to 10 meant a 15% decrease in reciprocity likelihood if they were women, but only 5% if they were men, a product of distinct effects of absolute BMI difference for each gender (*β*_women_ = − 0.08, *β*_men_ = − 0.04, *p* < 0.001). That is, women’s online connections were less likely to be reciprocated as they diverged in BMI, compared to men’s. We note that the interaction between absolute BMI difference and absolute age difference makes this gender distinction valid only when the absolute age difference was small, becoming nullified and even inverted at greater age difference values (Fig. 4). In other words, such gender differences in reciprocity likelihood occurred only when individuals were of similar age.

## Discussion

In this study, we examined patterns of age and BMI similarity among same-gender connections in an online social network. We found that age- and BMI-based homophily occurred in both men’s and women’s online reciprocal connections. Age and BMI homophily occurred somewhat more strongly among men than among women, albeit this difference was minute. We also found a negative association between absolute differences in age and BMI among users and the likelihood of reciprocity among individuals of both genders. In other words, as two users more strongly diverged in these traits, their online interaction was less likely to be reciprocated. This negative association was similar between men and women in respect to age, but was slightly stronger among women than men regarding BMI.

Our finding that age homophily occurs in an online social network from Slovakia should not be surprising, given that many studies reveal individuals associate with others of similar age (e.g., Kmetty et al., 2017; Smith et al., 2014). Age homophily may occur because same-aged individuals are more likely to share experiences and preferences (Adams & Blieszner, 1989). However, existing social and structural norms may also promote such a pattern. For instance, having inter-generational friends is not well-perceived in modern society (Williams & Nussbaum 2013). Furthermore, modern educational systems and workplaces form age-segregated spaces, fostering interactions among similarly aged individuals (Hagestad & Uhlenberg, 2006; Uhlenberg & De Jong Gierveld, 2004). If these processes influence real-life social ties and online connections reflect those, then even though users in the Pokec social network had the opportunity to contact others of different ages (producing close to zero expected by chance homophily coefficients in Fig. 2A, B), online networks should reveal age-based homophily (e.g., Mazur & Richards, 2011).

In respect to gender differences, we found that the difference between men and women in age assortativity was very small, with men showing slightly stronger similarity in age than women. These patterns might suggest that women’s tendencies to form relationships with allocare providers, who are often older and younger female kin (Crittenden & Marlowe, 2008; Ivey, 2000; Lancaster, 1971; Page et al., 2019), could result in lower age assortativity in female networks. However, because we could not ascertain whether online ties represented friends or family members, these speculations warrant further investigation. Moreover, there was no gender difference regarding the effect of age on the likelihood of reciprocity of online connections (Figs. 2C, 4). This indicates that men and women were equally likely to reciprocate connections with same-aged same-gender peers, despite our predictions of potential gender differences.

In comparison with age homophily, BMI homophily has been less often explored in extant research. Although some public health studies consider the effect of BMI in relationships, they are usually concerned with obesity propagation (e.g., Christakis & Fowler, 2007; De La Haye et al., 2011; but see also Goyal et al., 2014). The role of body size on friendships is more frequently discussed in the context of body image and body dissatisfaction (e.g., Gerner & Wilson, 2005; Kenny et al., 2018; Sharpe et al., 2014; Webb & Zimmer-Gembeck, 2014). This is because both girls/women (Grogan & Wainwright, 1996; Slevec & Tiggemann, 2011) and boys/men (e.g., Blond, 2008; Galioto & Crowther, 2013; McCabe & Ricciardelli, 2004) commonly express body dissatisfaction, which can impact their wellbeing by contributing to the development of unhealthy behaviors (Mintz & Betz, 1988; Vincent & McCabe, 2000) and depression (Barnes et al., 2020; Bornioli et al., 2021). Unfortunately, the data we used in the present study do not include measures of body image, restricting our ability to provide insights into this matter.

Unlike with age homophily, we observed wider gender differences in BMI-based homophily, albeit in inconsistent directions. For example, our results on assortativity show that BMI homophily is greater among men: men were more likely to show BMI-based homophily in their reciprocated ties than were women. In contrast, our results on tie reciprocity show that BMI-based homophily is greater among women: women were less likely to reciprocate a friendship when there was a greater difference between their BMI. These patterns might suggest that men show greater homophily in whom they chose to follow, whereas women show greater homophily in whom they choose to follow back (after being followed by a same-gender peer). However, because our data cannot provide insight into the mechanisms of the observed patterns, follow-up research is needed to adjudicate such possibilities. Although there are evolutionary (e.g., strong coalition partners; Eisenbruch et al., 2016; Hall, 2011; Reynolds & Palmer-Hague, 2022) and sociocultural (e.g., idealization of male muscularity; Frederick et al., 2005) explanations as to why men should exhibit greater BMI-based homophily, there are similar evolutionary (e.g., reducing mating rivalry; Bleske-Rechek & Lighthall, 2010; Bleske-Rechek et al., 2014) and sociocultural (e.g., discrimination against overweight individuals; Geen, 2014) explanations as to why women should be more likely to exhibit BMI-based homophily. Like the literature, our results conflict and thus, we do not have clear evidence to suggest that one gender clearly shows stronger homophily in BMI relative to the other. Because these patterns are countervailing, another possibility is that both men and women value homophily and that the genders do not differ in their desire to reciprocate friendships with individuals of similar body size.

We also emphasize that body size, including BMI, can be a meaningful characteristic for social status, but for slightly different reasons depending on gender (Buss et al., 2020). Women often seek a thin body that is deemed attractive (Swami & Tovée, 2005; Tovée & Cornelissen, 2001; Tovée et al., 1998) which can be exacerbated by media depictions (Grabe et al. 2008). Conversely, many men desire a muscular body for themselves in hopes of attaining the idealized male body image propagated by the media (Agliata & Tantleff-Dunn, 2004; Barlett et al., 2008; McCreary et al., 2005), despite women preferring men with ordinary bodies (Pope et al., 2000). The existence of idealized figures for women and men thus could influence their same-gender friendships. Hence, the extent that individuals consider others’ body size to initiate and maintain friendships needs to be further explored in detail in future studies.

The literature on friendships is diverse (reviewed in Dunbar, 2018), but many studies contend that gender differences in same-gender friendships reflect gender-specific adaptations regarding friendship decision-making (e.g., Ayers et al., 2023; Eisenbruch & Roney, 2020; Krems & Conroy-Beam, 2020; Vigil, 2007; Williams et al., 2022). For example, many investigations expect directional friendship preferences for certain traits among both men (e.g., physically formidable men might be advantageous for hunting and war; Eisenbruch & Roney, 2020) and women (e.g., nurturing women might be advantageous for alloparenting; Williams et al., 2022). Although this rationale is plausible, it might not sufficiently address how friendships develop when potential partners diverge in traits. For example, John’s high status may elicit Ben’s interest in being his friend, but why would John want to befriend Ben if he is a lesser status man? Widespread directional preferences should suggest that people with less desirable attributes would often fail in their friendship attempts. Krems and Conroy-Beam (2020) showed that people settle for less and prefer others of similar “friendship value” to their own to circumvent this. However, another interpretation of their results is that people seek similar individuals to befriend, not because they are doing the best of a bad job, but because similarity itself promotes connection. Moreover, friendship preferences might differ from actual friendship interest after interaction (Huang et al., 2020), indicating that researchers might benefit from using varied approaches to assess friendship formation. Here, we demonstrate that homophily matters in the formation of social ties, and thus encourage researchers to incorporate this pattern in further studies and theorizing.

Our study possesses two noteworthy strengths: a large sample size and the examination of existing ties (rather than self-reported preferences). Although these allowed greater power and insight into ongoing relationships, our reliance on secondary data restricted the information we could extract from the network and its users. First, we were unable to verify the intensity and the nature of the relationships between users. It is possible that some factors may only mediate certain relationships (e.g., greater concern over body types among more close than distant friends), meaning that our results should be taken with caution. Second, we do not know how missing and erroneous data might have affected our results. There is evidence that people intentionally misrepresent themselves on online social networks to make themselves more appealing to potential mates (Toma et al., 2008). If some users were more likely than others to omit or lie about their personal information (e.g., fear of not conforming to societal standards leading to underreporting weight), then our results may be less reliable. This may be the case as less than 3% of women in our dataset reported they were overweight (BMI > 25, see Fig. 1), despite 15.7% of adult women in the Slovakian population being obese during data collection (Eurostat, 2011). Third, BMI is a crude metric of body proportions and there are likely more nuanced body measurements that can more strongly determine mating outcomes and social status (e.g., muscularity, abdominal depth, localized fat, waist girth; Barlev et al., 2022; Bogin and Varela- Silva, 2012; Brooks et al., 2015; Frederick & Haselton, 2007; Goyal et al., 2014; Krems & Bock, 2023; Rilling et al., 2009). For example, some research suggests women experience greater stigma when their bodies deposit fat across the stomach compared to the hips and thighs (Krems & Neuberg, 2022). Other work finds that magazines targeting men idealize more muscular bodies than those targeting women (Frederick et al., 2005). Our crude measure of body size (BMI) could not partial out effects due to body shape or muscularity (versus fat), thereby limiting our understanding of these more nuanced and likely gender-differentiated associations. Future research might therefore incorporate a more diverse array of body assessments to address this limitation.

Fourth, our investigation relied on existing data, so we could not assess users’ motivations for their followership patterns. For example, we cannot ascertain whether a thinner or heavier female peer is less likely to reciprocate a connection with a woman of a different weight due to perceived mating rivalry or social competition. Fifth, even though we assume that online social connections may shed some light on offline relationship patterns, our data cannot confirm the degree to which this is true. For example, people may follow others online for reasons that diverge from offline social tendencies, such as entertainment, shock, or inspiration. Thus, future investigations could employ other methods to examine whether the patterns observed here extend to offline friendships. These might include surveys using friend dyads (as in Bleske-Rechek & Lighthall, 2010) or small networks in which details of relationships can be better captured (e.g., classrooms).

Last, we described our findings as gender differences because our data comprise of users’ self-selected identification. Although we would expect patterns to reflect sex differences if the majority of the population is cisgender, we do not have any way of assessing biological sex. Future research could address this drawback by measuring both sex assigned at birth and gender identification to better examine whether asymmetries in relationship patterns reflect sex or gender differences.

### Supplementary Information

Below is the link to the electronic supplementary material.

Supplementary file1 (DOCX 734 kb)

## Data Availability

The Pokec dataset is publicly available at https://snap.stanford.edu/data/soc-Pokec.html.

## Code Availability

Processed data and analysis scripts are available at https://osf.io/2x3nu/?view_only=da44e04e5845432a8cb4fbb5c28db145. All authors gave final approval for publication.
