# Correction: Exploring Within-Gender Differences in Friendships Using an Online Social Network

**DOI:** 10.1007/s10508-024-02973-8

**Published:** 2024-08-06

**Authors:** Pietro Pollo, Tania A. Reynolds, Khandis R. Blake, Michael M. Kasumovic

**Affiliations:** 1 Evolution and Ecology Research Centre, School of Biological, Earth and Environmental Sciences, University of New South Wales, 5 Floor, Building E26, Kensington, NSW 2052 Australia; 2 Department of Psychology, University of New Mexico, Albuquerque, NM USA; 3 Melbourne School of Psychological Sciences, The University of Melbourne, Melbourne, VIC Australia

**Correction to: Archives of Sexual Behavior** 10.1007/s10508-024-02906-5

Due to an error during production, the word “sex” was erroneously changed to “gender” in six places in the text of the originally published version of the article.

The original article has been corrected.

