# Supplementary material for: Exploring Within-Gender Differences in Friendships Using an Online Social Network
Source: Arch Sex Behav. 2024 Jun 11;53(8):3187–201. doi: 10.1007/s10508-024-02906-5 (PMC11335865; doi:10.1007/s10508-024-02906-5)
Supplement: Supplementary file 1 — Supplementary file1 (DOCX 734 kb) [file 10508_2024_2906_MOESM1_ESM.docx]

**Appendix I**


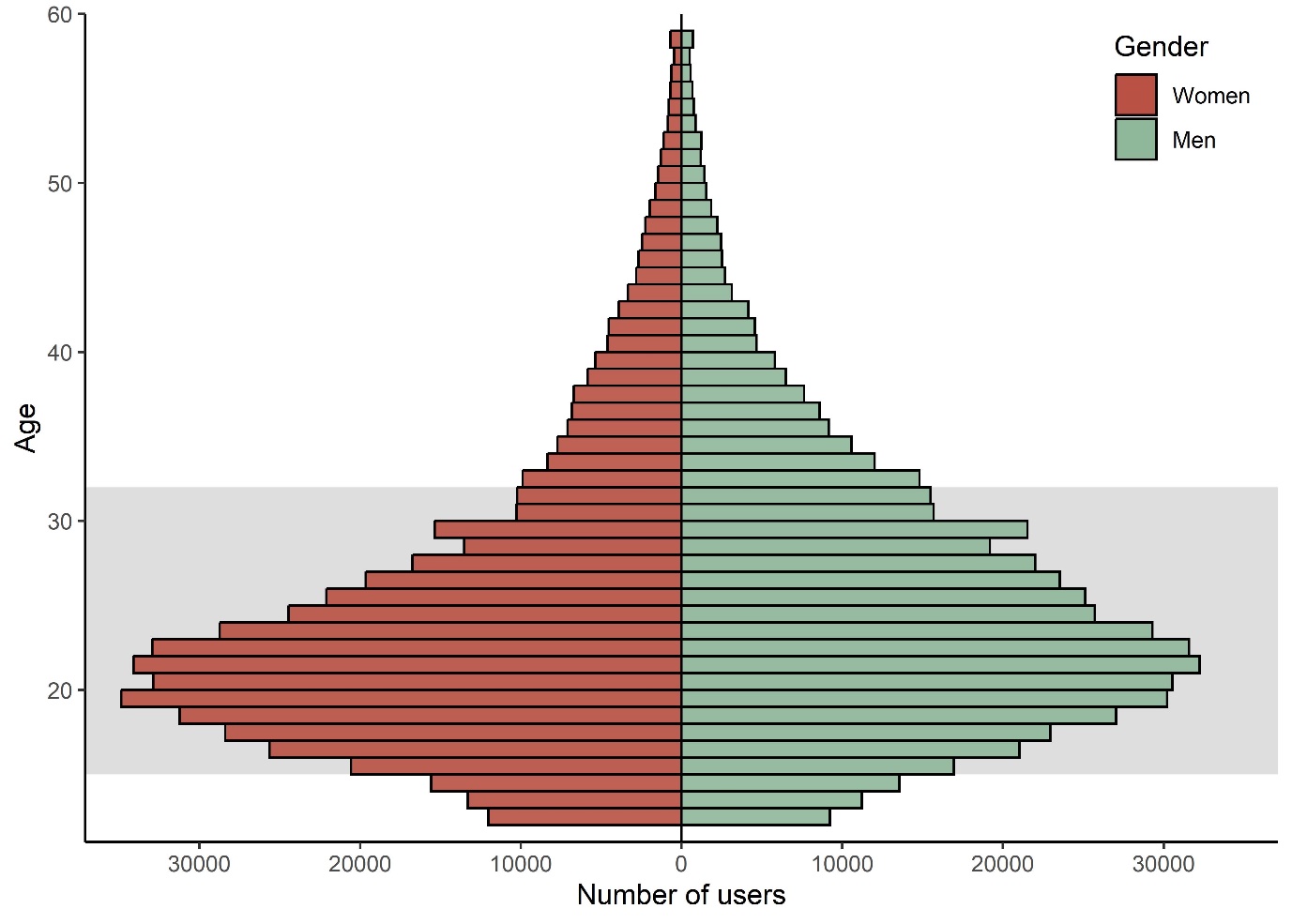


Figure S1. Users’ age distribution according to gender. Grey area highlights the subset of users used for our analyses (15-32 years old).


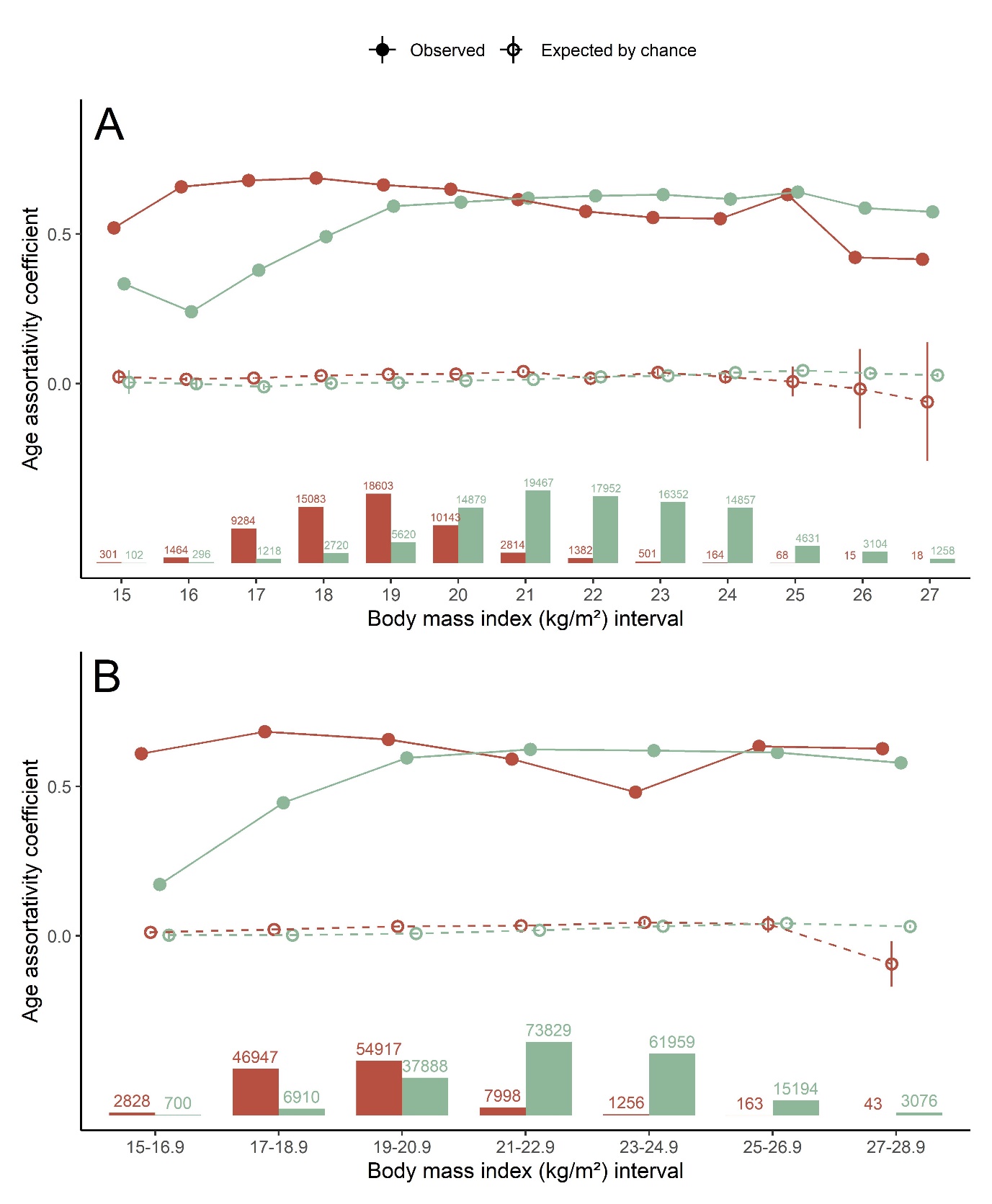


Figure S2. Age assortativity coefficients observed and expected by chance in subsets of the network using BMI intervals of one (A) or two (B) units each. Whiskers represent the 95% coefficient interval for mean assortativity coefficients (only for the ones expected by chance). Bars represent the number of observed connections in each subset of the network.


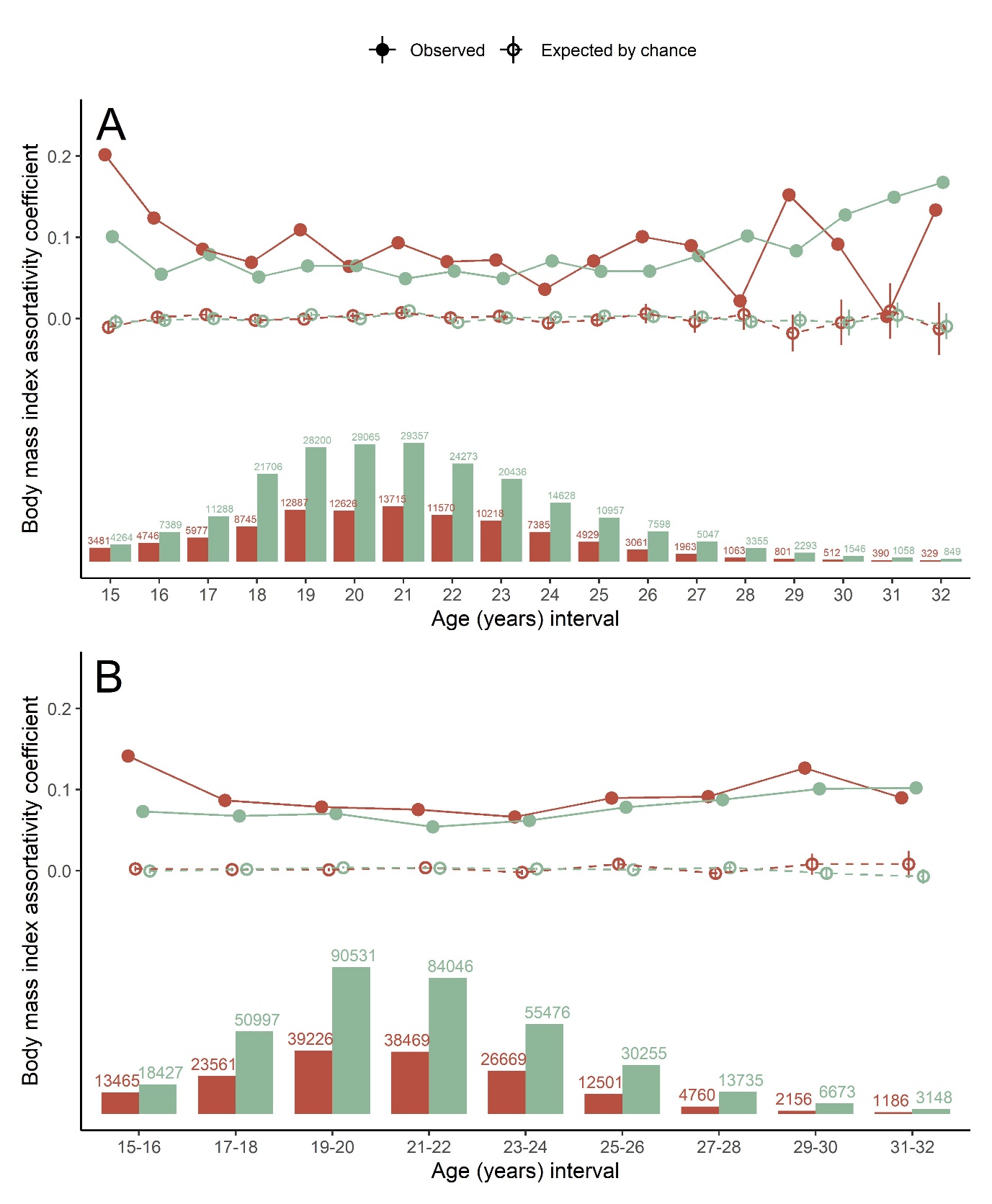


Figure S3. BMI assortativity coefficients observed and expected by chance in subsets of the network using age intervals of one (A) or two (B) years each. Whiskers represent the 95% coefficient interval for mean assortativity coefficients (only for the ones expected by chance). Bars represent the number of observed connections in each subset of the network.
